# Autoregulatory-guided management in traumatic brain injury: does age matter?

**DOI:** 10.1007/s00701-025-06474-y

**Published:** 2025-02-28

**Authors:** Teodor Svedung Wettervik, Erta Beqiri, Anders Hånell, Stefan Yu Bögli, Ihsane Olakorede, Xuhang Chen, Adel Helmy, Andrea Lavinio, Peter J. Hutchinson, Peter Smielewski

**Affiliations:** 1 Department of Medical Sciences, Section of Neurosurgery, Uppsala University, 751 85 Uppsala, Sweden; 2 Brain Physics Laboratory, Department of Clinical Neurosciences, Division of Neurosurgery, University of Cambridge, Cambridge, UK; 3 Department of Clinical Neurosciences, Division of Neurosurgery, Addenbrooke’s Hospital, University of Cambridge, Cambridge, UK; 4 Neurosciences and Trauma Critical Care Unit, Addenbrooke’s Hospital, Cambridge University Hospitals, Cambridge, UK

**Keywords:** Age, Neurocritical care, Optimal cerebral perfusion pressure, Pressure reactivity index, Traumatic brain injury

## Abstract

**Background:**

Although older traumatic brain injury (TBI) patients often exhibit cerebral autoregulatory impairment with high pressure reactivity index (PRx), the role of autoregulatory-guided management in these patients remains elusive. In this study, we aimed to explore if age affected the prognostic role of the autoregulatory variables, PRx and the PRx-derived optimal cerebral perfusion pressure (CPPopt), in a large TBI cohort.

**Methods:**

In this observational study, 550 TBI patients who had been treated in the neurocritical care unit, Addenbrooke’s Hospital, Cambridge, UK, between 2002 and 2022 with available data on age, intracranial pressure monitoring, and outcome (Glasgow Outcome Scale [GOS]) were included. The patients were classified into three age groups; youth and early adulthood (16–39 years), middle adulthood (40–59 years), and senior adulthood (60 years and above). Autoregulatory variables were studied in relation to outcome using heatmaps. Multivariate logistic regressions of mortality and favourable outcome (GOS 4 to 5) were performed with PRx and ΔCPPopt (CPP-CPPopt) in addition to baseline variables.

**Results:**

TBI patients in the senior adulthood group exhibited higher PRx and lower ICP than younger patients. There was a transition towards worse outcome with higher PRx in heatmaps for all age groups. The combination of high PRx together with low CPP or negative ΔCPPopt was particularly associated with lower GOS. In multivariate logistic regressions, higher PRx remained independently associated with higher mortality and lower rate of favourable outcome in the senior adulthood cohort. There was a transition towards worse outcome for negative ΔCPPopt for all age groups, but it did not reach statistical significance for the senior adulthood group.

**Conclusions:**

PRx was found to be an independent outcome predictor and influenced the safe and dangerous CPP and ΔCPPopt interval for all age groups. Thus, TBI patients older than 60 years may also benefit from autoregulatory-guided management and should not necessarily be excluded from future trials on such therapeutic strategies.

**Supplementary Information:**

The online version contains supplementary material available at 10.1007/s00701-025-06474-y.

## Introduction

Cerebral autoregulation (CA)-guided management has emerged as a promising concept to optimize cerebral blood flow (CBF) in traumatic brain injury (TBI) patients requiring intracranial pressure (ICP)-therapy in the neurocritical care unit (NCCU) [28, 34]. This idea is based on the fact that the homeostatic cerebral vasoreactivity aimed at maintaining CBF normal over a broad range of cerebral perfusion pressures (CPP) often becomes impaired in TBI during the acute phase [16]. Consequently, these patients may develop ischaemia or hyperaemia, even if their CPP is kept within the fixed target interval according to the current guideline recommendations [5, 6, 24, 26]. The pressure reactivity index (PRx) – as a surrogate measure for CA – and the derived optimal CPP (CPPPopt) – corresponding to the lowest and thus best PRx value – are the most validated tools for continuous CA monitoring and potential treatment guidance [2, 8, 34]. Previous studies have consistently demonstrated that elevated PRx and CPP deviation from CPPopt (ΔCPPopt) are strongly associated with worse outcome [2, 8, 22]. Integrating PRx and CPPopt into clinical management could potentially improve CBF and patient recovery after TBI [17, 34]. Recently, a phase II-trial demonstrated that targeting CPPopt was both safe and feasible [28]. The next step is to design a phase III-trial to determine if CA-guided management translates into improved functional outcomes. A key issue in these attempts is to determine the optimal patient selection strategy for such a study. In particular, elderly patients are generally excluded from clinical trials. This is partly due to significantly lower expectations of treatment effects and partly because higher age statistically coincides with increased co-morbidities, leading to issues with heterogenous study cohorts [10].

However, there is currently a paradigm shift in TBI. This pathology has transitioned from a disease characterized by young patients involved in road traffic accidents, to elderly, frail, patients who sustained head injuries after falls [23]. Furthermore, these old patients often have acquired arterial hypertension and cerebrovascular diseases, which are expected to influence their CA function and make them more susceptible to CBF disturbances and secondary brain injury [13]. Arguably, these patients would therefore be particularly suitable for personalized medicine approaches using CA-guided therapy [2, 19, 34]. Nevertheless, a recent study from Uppsala raised concerns with this idea. In that study, higher PRx, was only weakly associated with mortality and CPP deviation from CPPopt was not related to outcome in an elderly TBI cohort [15]. In outcome heatmaps of the same study, while PRx was useful to indicate the detrimental effect of exceeding both the lower (high PRx with low CPP or negative ΔCPPopt) and upper (high PRx with high CPP or positive ΔCPPopt) limit of CA among younger patients, it had no such effect in the older TBI cohort. It was speculated whether the primary brain injury itself was of greater prognostic value, while secondary pathophysiological mechanisms such as CA impairment only had a limited impact on outcome in older patients. However, generalisations of single-centre studies are often limited and confirmatory studies in other centres’ TBI cohorts are warranted. This is important, since the available evidence will influence the design of future trials of CA-guided management. Taking into account the epidemiological rise in elderly TBI patients, excluding them from these trials would clearly limit the potential use of such treatment strategies. Thus, the aim of this study was to explore the CA variables PRx and ΔCPPopt in relation to outcome for different age categories in another large cohort of adult TBI patients. In spite of the findings in the Uppsala cohort [15], we hypothesized that these CA variables would remain associated with outcome also among older patients, since their frailty and reduced physiological and functional reserve would make them susceptible to CA impairment.

## Materials and methods

### Patients and study design

In this observational, single-centre study, 781 TBI patients, aged ≥ 16 years, with ICP-monitoring, who had been treated at the NCCU, Addenbrooke’s Hospital, Cambridge, UK between 2002 and 2022 were eligible for inclusion. Those with missing information on age (n = 3), outcome (n = 205), and with less than 12 h of ICP monitoring time (n = 23) were excluded. The final study cohort included 550 patients. The patients were classified into three age groups; youth and early adulthood (16–39 years), middle adulthood (40–59 years), and senior adulthood (60 years and above). These age classes were consistent with previous studies [14] and pragmatic, since in the Brain Physics research database the age had been expressed with 10-year granularity for the purpose of de-identification.

### Management protocol

The management protocol has been described in previous studies [9]. The protocol underwent slight changes during the period of 20 years [9]. In brief, CPP was targeted above 60 mmHg, ICP below 20 to 25 (before barbiturates and decompressive craniectomy) mmHg, partial brain tissue oxygen (pbtO_2_) above 15 to 20 mmHg, partial pressure of carbon dioxide within 4.5–5 kPa, and arterial glucose within 6 to 8 mmol/L. PRx and CPPopt were available bedside since 2002 and 2012, respectively, and could be targeted based on the discretion of the treating doctor.

### Outcome

Clinical outcome was evaluated using GOS assessments six months post-TBI, either by clinical follow-up or telephone interviews [29, 32]. Outcome was dichotomized as survival vs. mortality and favourable vs. unfavourable functional recovery (GOS moderate disability and good recovery vs. death, vegetative, and severe disability).

### Collection and processing of cerebral physiological data

ICP was primarily monitored with intraparenchymal probes (Codman ICP MicroSensor, Codman & Shurtleff, Raynham, Massachusetts) via the Cambridge Cranial Access Device, but occasionally with an external ventricular drain. Arterial blood pressure (ABP) was monitored in the radial or femoral artery (Baxter Healthcare, Deerfield, Illinois) at the level of the heart (until 2015) or the foramen of Monro (2015 and forward). Physiological data were streamed at 50 to 250 Hz from the monitors to a laptop with the ICM + software (ICM + software, Cambridge Enterprise Ltd, University of Cambridge, UK) (https://icmplus.neurosurg.cam.ac.uk), which provided data integration, storage, and de-identification for the Brain Physics database. The physiological data were curated partially manually (annotation of bad sections of data) and partially automatically (using heuristic algorithms) and subsequently down-sampled to 10 s-values by coarse-graining. The good monitoring time (GMT) was defined as the time remaining after excluding of these artefacts and time outside the NCCU due to surgery and imaging. PRx was calculated as the moving Pearson correlation coefficient of 30 consecutive 10-s average values of ABP and ICP [8, 35]. CPPopt was calculated according to the multi-window weighted algorithm based on a data buffer of 2 to 8 h and defined as the corresponding CPP with the lowest PRx [4], i.e., the same algorithm that was used in the COGiTATE trial [28]. ∆CPPopt was defined as the difference between actual CPP and CPPopt. Minute-by-minute average values of all variables were obtained and considered for the next analysis. The median values of ICP, PRx, CPP, and CPPopt, were calculated over the GMT. Furthermore, the %GMT of PRx above + 0.20, ΔCPPopt < −5 mmHg, and ΔCPPopt >  + 5 mmHg were calculated. The PRx threshold at + 0.20 was chosen, as this level has previously been investigated for assessment of the limits of working CA, and performed similarly to higher thresholds [5]. The ΔCPPopt-thresholds below −5 mmHg and above + 5 mmHg were chosen in accordance with the treatment target concepts of the COGiTATE trial [28].

### Visualization of outcome

The association between PRx and ΔCPPopt in relation to outcome (GOS) were visualized based on single- and two-variable analyses with R-scripts used in previous studies [25, 27]. The purpose of the single-variable analysis was to visualize the transition between outcomes for individual physiological variables [25], while the two-variable plots were created to determine if the CA status (PRx) interacted with ΔCPPopt or CPP in relation to outcome. In the first approach of single variables [25], an outcome correlations heatmap was created based on PRx and ΔCPPopt, respectively, divided into separate grid cells. PRx was divided into 20 grid cells (range −1.00 to + 1.00, 0.10 intervals) and ΔCPPopt into 20 grid cells (range −40 to + 40 mmHg, 4 mmHg intervals). In the second approach of two variables, an outcome heatmap was created in a similar way, but based on combinations of PRx/ΔCPPopt and PRx/CPP. The PRx/ΔCPPopt plot was based on 400 grid cells (20*20) with the same ranges and intervals for each variable as in the single-variable heatmap. The PRx/CPP plot was also based on 400 grid cells (20*20), with the same PRx range/interval as previously while CPP was divided into 4 mmHg-intervals over the 40–120 mmHg-range.

After defining the coordinate grid of these maps, the %GMT was calculated for each patient for every grid cell. Similar to a previous study [25], the data within each grid cell was dichotomized with respect to both GOS and %GMT before calculating the phi (Pearson correlation of binary variables) coefficient [18]. The phi coefficient was chosen as a metric as it is simple and easy to interpret and both indicates correlation strength and direction. Since the choice of dichotomization point was not obvious, all possible values were evaluated, as long as they resulted in at least 5 patients on each side of the split. The strongest correlation (highest absolute value) for each grid cell, with a corresponding GOS and %GMT dichotomization cut-off, was used, a strategy referred to as “optimized dichotomy” [25]. This resulted in a single correlation value for each grid cell. To produce smoother images, each grid cell was divided into 3 * 3 sub grid cells followed by application of a Gaussian kernel filter (standard deviation of 2 grid cells). The final correlation values were visualized using the jet colour scale (blue corresponding to favourable and red to unfavourable). The colour scale was limited to correlations within ± 0.50 and results from grid cells with less than 5 patients that had at least 5 min of monitoring time were coloured as white.

In addition to these outcome visualizations, complementary density heatmaps were created by counting the number of observations within each grid cell and dividing it by the highest count among all grid cells. A similar smoothing process was applied and the density heatmaps were then coded using the jet colour scale (blue corresponding to frequent and red to rare). Lastly, the dichotomization points in GOS and %GMT as well as the percentage of patients below the GMT dichotomization point were visualized in analogous plots after colour-coding and similar smoothing processes.

### Statistical analysis

Descriptive data were presented as numbers (proportions) or medians (interquartile range [IQR]). Differences in demography, admission variables, cerebral physiology, treatments, and outcome among the age groups were evaluated with Kruskal–Wallis and chi-square tests, depending on the type of data. The univariate association between autoregulatory insults (%GMT of PRx >  + 0.20, ΔCPPopt < −5 mmHg, and ΔCPPopt > 5 mmHg) with GOS was evaluated with the Spearman rank correlation test for each age category. Multivariate logistic regression analyses with mortality and favourable outcome, respectively, as dependent variables were performed. In these regressions, the %GMT of PRx >  + 0.20 and ΔCPPopt < −5 mmHg in addition to Glasgow Coma Scale (GCS) and ICP as baseline variables of injury severity were used as independent variables. ΔCPPopt < −5 mmHg was used as an CPPopt-insult rather than ΔCPPopt >  + 5 mmHg in these regressions since the former was more strongly associated with lower GOS in all cases. A p-value below 0.05 was considered statistically significant. The statistical analyses were conducted in R software (version 4.4.1) [1].

## Results

### Demography, injury severity, treatments, and outcome

As described in Table 1, of 550 TBI patients, 275 patients were in the youth and early adulthood group, 170 in the middle adulthood group, and 105 were in the senior adulthood group. In brief, the male/female ratio, GCS, pupillary reactivity, and the treatment rate with decompressive craniectomy were similar among all three age groups. However, GOS and the rate of favourable outcome was lower, while mortality was higher, in the cohort of patients in the senior adulthood group. The proportion of senior adult TBI patients increased significantly from 16 to 23% between the first (2002–2011) and last (2012–2022) decades of the study (*p* < 0.01). The rate of favorable outcomes remained stable, at 50% compared to 47% (*p* = 0.53).

**Table 1:** Demography, injury severity, treatments, and outcome for each age group

Variables	Young	Middle age	Old	*p*-value
Patients	275	170	105	NA
Age (years)	25 (25–35)	45 (45–55)	65 (65–75)	NA
Sex (male/female)	206/50 (80/20%)	104/39 (73/27%)	68/18 (79/21%)	0.19
GCS	7 (3–9)	7 (4–9)	7 (4–10)	0.40
Pupillary reactivity (intact/1 unreactive/2 unreactive)	118/19/6 (83/13/4)	64/12/2 (82/15/3%)	51/5/2 (88/9/3%)	0.78
Decompressive craniectomy	83 (33%)	44 (29%)	22 (25%)	0.27
GOS	4 (3–4)	3 (2–4)	3 (1–4)	** < *0.001***
Favourable outcome	160 (58%)	76 (45%)	32 (30%)	** < *0.001***
Mortality	45 (16%)	37 (22%)	47 (45%)	** < *0.001***

Missing data: sex (*n* = 65), GCS (*n* = 51), pupillary reactivity (*n* = 271), decompressive craniectomy (*n* = 61). Continuous/ordinal variables are described as medians (IQR) and categorical variables as numbers (proportions)

*GOS *Glasgow Outcome Scale, *IQR *Interquartile range, *NA *Not applicable

### Cerebral physiology – differences among the age groups

As demonstrated in Table 2, the senior adulthood patients exhibited lower median ICP than the youth and early adulthood patients, while median PRx was higher. Median CPPopt was higher in the middle adulthood group compared to the youth and early adulthood group, but was comparable to the senior adulthood patients. The %GMT of ΔCPPopt below −5 mmHg and above + 5 mmHg was similar among all age groups. The cerebral physiological variables for each age group were also analysed before and after the change in reference point of ABP (heart level until the end of 2014 and foramen of Monro from 2015; Supplementary Table 1). In brief, for all age groups, there were consistent trends towards lower median ICP, CPP, and CPPopt, while PRx remained unchanged.

**Table 2:** Cerebral physiological variables during neurocritical care

Variables	Young	Middle age	Old	p-value
ICP (mmHg)	14 (11–18)^a^	14 (10–17)^a,b^	12 (8–16)^b^	***0.001***
CPP (mmHg)	75 (72–78)^a^	77 (73–81)^b^	75 (71–81)^a,b^	***0.01***
PRx (coefficient)	+ 0.01 (−0.09- + 0.12)^a^	+ 0.10 (−0.04- + 0.22)^b^	+ 0.10 (−0.04- + 0.24)^b^	< ***0.001***
PRx > + 0.20	32 (23–43)^a^	40 (28–52)^b^	40 (30–53)^b^	< ***0.001***
CPPopt (mmHg)	75 (71–79)^a^	77 (73–82)^b^	75 (72–81)^a,b^	***0.01***
ΔCPPopt < −5 mmHg	26 (20–33)	28 (21–36)	28 (20–40)	0.26
ΔCPPopt > + 5 mmHg	33 (25–41)	33 (26–41)	32 (24–41)	0.80

Different superscripted letters were used to indicate statistical differences between the groups, after Kruskal–Wallis test with subsequent post-hoc Bonferroni-adjusted analysis. If two groups were statistically different, but both overlapped with a third group, “^a,b^” was written to indicate that the third group overlapped with both of the other groups. Bold and italics indicate statistical significance

*CPP *Cerebral perfusion pressure, *CPPopt *Optimal CPP, *ICP *Intracranial pressure, *PRx *Pressure reactivity index

### Autoregulatory insults in relation to outcome among the age groups

As demonstrated in Table 3, higher %GMT of PRx above + 0.20 was associated with lower GOS (r = −0.30, p < 0.01) in the senior adulthood cohort, but no significant association was found between outcome and %GMT of ΔCPPopt < −5 mmHg or ΔCPPopt >  + 5 mmHg in this group. In the middle adulthood cohort, higher %GMT of ΔCPPopt < −5 mmHg (r = −0.18, p < 0.05), but neither PRx >  + 0.20 nor ΔCPPopt >  + 5 mmHg were associated with GOS. In the youth and early adulthood cohort, PRx >  + 0.20 (r = −0.35, p < 0.001) and ΔCPPopt < −5 mmHg (r = −0.23, p < 0.001) were associated with lower GOS, while ΔCPPopt >  + 5 mmHg (r = 0.22, p < 0.001) had the opposite association.

**Table 3:** Autoregulatory insults in relation to GOS – a Spearman correlation analysis

Variables	Young	Middle age	Old
PRx > + 0.20	***−0.35***^***c***^	−0.14	***−0.30***^***b***^
ΔCPPopt < −5 mmHg	***−0.23***^***c***^	***−0.18***^***a***^	−0.11
ΔCPPopt > + 5 mmHg	***0.22***^***c***^	0.07	0.18

The table displays the correlation coefficients between %GMT below/above certain intervals of the autoregulatory variables in relation to GOS. Superscripted letters indicate statistical significance; ^a^p < 0.05, ^b^p < 0.01, ^c^p < 0.001, which all were highlighted with bold and italics

*CPPopt *Optimal cerebral perfusion pressure, *GMT *Good monitoring time, *GOS *Glasgow Outcome Scale, *PRx *Pressure reactivity index

In multivariate logistic outcome regressions, higher %GMT of PRx >  + 0.20 was independently associated with favourable outcome in both the youth and early adulthood and the senior adulthood cohorts and in all cohorts regarding mortality (Table 4). Furthermore, higher %GMT of ΔCPPopt < −5 mmHg was independently associated with mortality in the young and middle adulthood group.

**Table 4:** Autoregulatory insults in relation to favourable outcome and mortality – multivariate logistic outcome regressions for separate age groups

	Favourable outcome			Mortality		
	Young	Middle age	Old	Young	Middle age	Old
PRx > + 0.20	***0.97 (0.95–0.99)***^***c***^	0.99 (0.97–1.01)	***0.97 (0.94–1.00)***^***a***^	***1.05 (1.03–1.08)***^***c***^	***1.03 (1.00–1.06)***^***a***^	***1.04 (1.01–1.07)***^***b***^
ΔCPPopt < −5 mmHg	0.99 (0.96–1.01)	1.00 (0.97–1.02)	1.00 (0.97–1.04)	***1.07 (1.03–1.11)***^***c***^	***1.04 (1.01–1.09)***^***a***^	1.00 (0.97–1.03)

In addition to %GMT of PRx >  + 0.20 and ΔCPPopt < −5 mmHg, GCS and median ICP were used as baseline variables in the regressions. The table indicates the OR with their corresponding 95% CI for the autoregulatory variables. Superscripted letters indicate statistical significance; ^a^p < 0.05, ^b^p < 0.01, ^c^p < 0.001, which all were highlighted with bold and italics

*CI *Confidence interval, *CPPopt *Optimal cerebral perfusion pressure, *OR *Odds ratio, *PRx *Pressure reactivity index

**Fig. 1:**
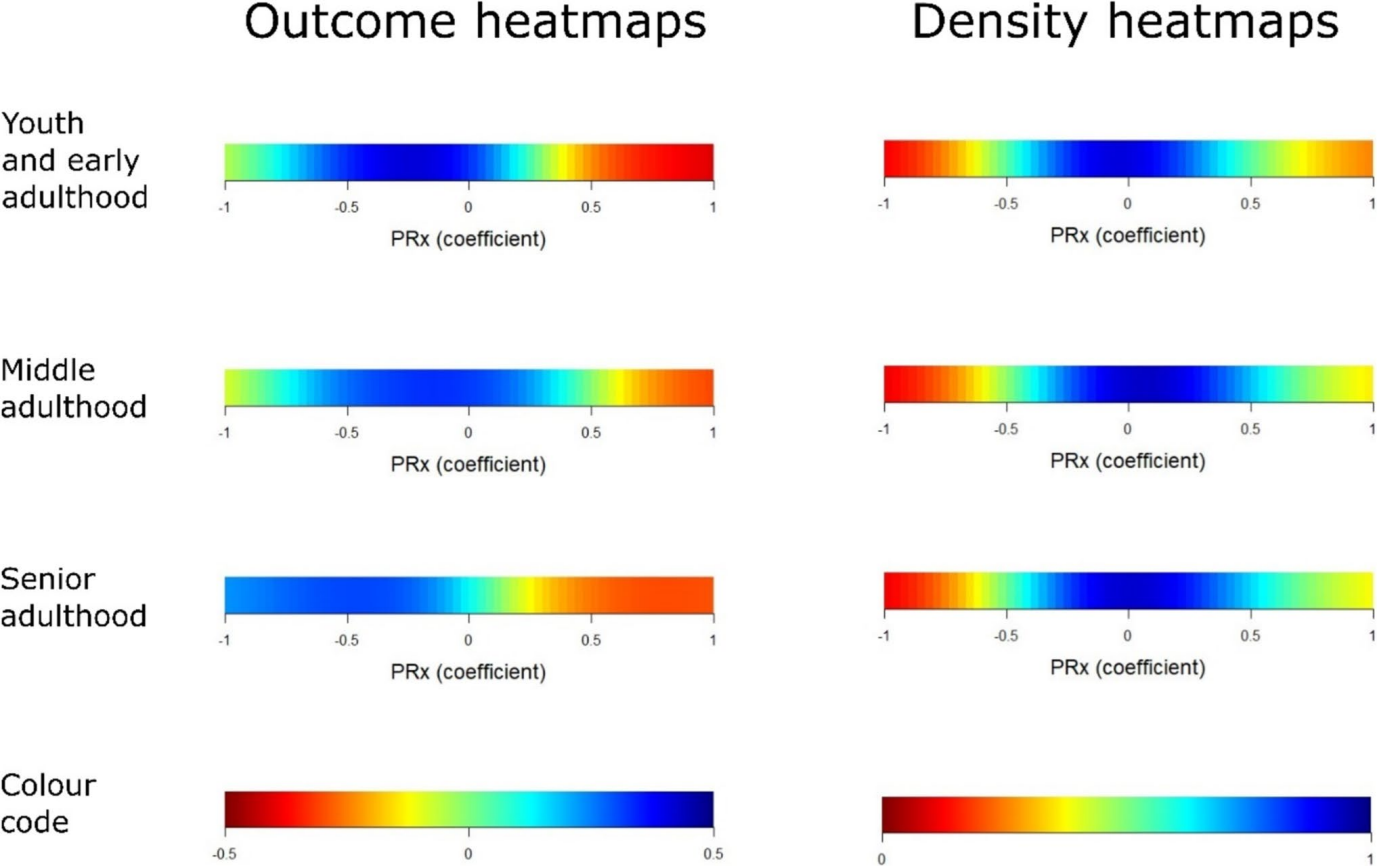
PRx – outcome and density heatmaps for all age groups. ***Outcome heatmaps*** – These figures show the correlation between %GMT of PRx within certain intervals in relation to outcome. As illustrated, with slight variations, there was a consistent transition towards worse outcome for higher PRx for all age groups. ***Density heatmaps*** – These figures show the data distribution over the entire PRx range for all age groups. As illustrated, PRx was centred around 0 ± 0.50. GMT = Good monitoring time. PRx = Pressure reactivity index

**Fig. 2:**
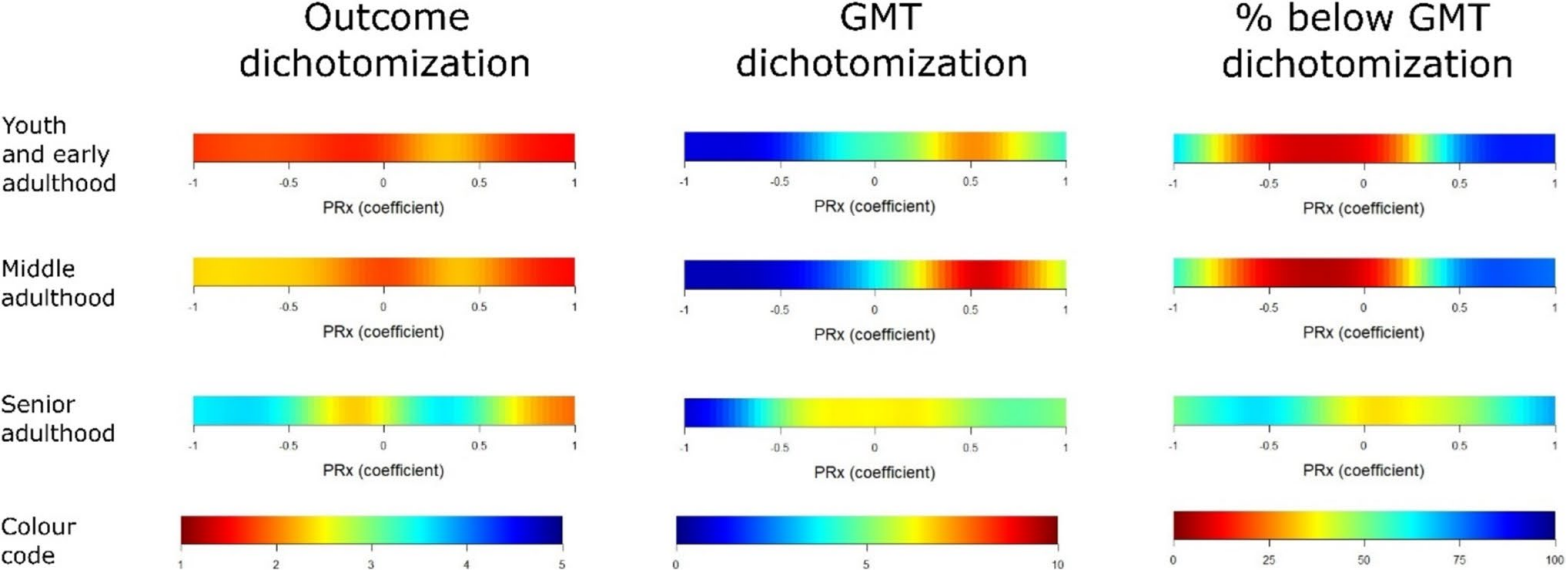
Dichotomizations points in the single-variable %GMT analysis of PRx. The outcome heatmaps in Fig. 1 show the colour-coded correlation coefficients based the “optimized dichotomy”. These heatmaps show the corresponding heatmaps that demonstrate the dichotomization points in terms of outcome (GOS) and percentage of monitoring time (GMT) over the PRx range with multiple grid cells of different intervals. ***Outcome dichotomization*** – these heatmaps illustrate the dichotomization point in GOS for PRx. As illustrated, the dichotomization typically occurred for mortality (GOS = 1) over the entire PRx range for the youth and early adulthood and the middle adulthood group, while it also occurred around GOS 3 for the senior adulthood group for some PRx intervals. ***GMT dichotomization*** – these heatmaps illustrate the dichotomization point in %GMT for PRx. As illustrated, the dichotomizations were typically made when the patients spent a relatively smaller (blue < 5%) %GMT for negative PRx and a relatively larger (red ≈ 7–10%) %GMT for higher PRx (around + 0.50) in the youth and early adulthood and the middle adulthood group. In the senior adulthood group, the GMT dichotomization point also occurred at relatively small %GMT for negative PRx, but at more moderate %GMT (yellow/green ≈ 5%) for higher PRx. ***% below GMT dichotomization*** – these heatmaps illustrate the percentage of patients below the GMT dichotomization point for PRx. As illustrated, most patients were below the GMT dichotomization threshold for high values such as PRx above + 0.50 in the youth and early adulthood and the middle adulthood group, while it was distributed around 50% throughout the entire PRx-range in the senior adulthood group. GMT = Good monitoring time. GOS = Glasgow Outcome Scale. PRx = Pressure reactivity index

**Fig. 3:**
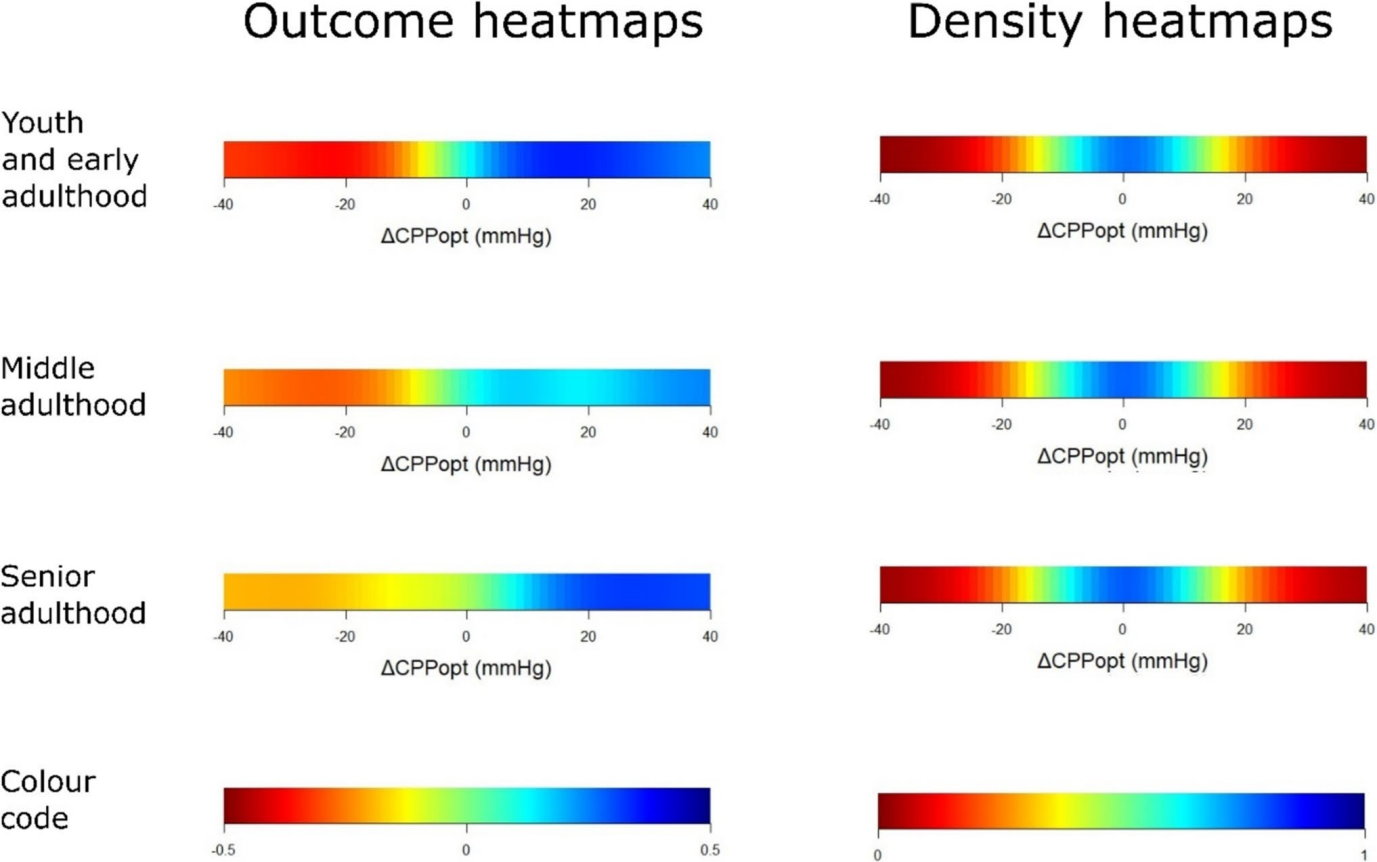
ΔCPPopt – outcome and density heatmaps for all age groups. ***Outcome heatmaps*** – These figures show the correlation between the %GMT of ΔCPPopt within certain intervals in relation to outcome. As illustrated, there was a trend towards worse outcome for negative ΔCPPopt for all age groups. ***Density heatmaps*** – These figures show the data distribution over a large ΔCPPopt range for all age groups. As illustrated, ΔCPPopt was centred around 0 ± 20 mmHg. CPP = Cerebral perfusion pressure. CPPopt = Optimal CPP. ΔCPPopt = Actual CPP – CPPopt. GMT = Good monitoring time

In outcome heatmaps, similar to the statistical findings in Table 3, there was a change from better to worse outcome for higher PRx and more negative ΔCPPopt in all age groups (Figs. 1, 2, and 3). In Fig. 1, there was a transition towards worse outcome when PRx exceeded approximately + 0.25 in the senior adulthood group, but this transition started slightly higher in the early youth and young adulthood and the middle adulthood groups. The “optimized dichotomy” occurred mostly between mortality and survival, the optimal GMT dichotomization was relatively high (10%) for elevated PRx, and most patients (more than 80%) were below that GMT dichotomization in the young and middle adulthood group (Fig. 2). These explanatory heatmaps indicate that PRx had the strongest outcome association when the subgroup who died and exhibited a higher GMT burden with elevated PRx was analysed in relation to the remaining patients. The dichotomization pattern was similar, but, e.g., the outcome dichotomization mostly occurred at a higher GOS (around 3) in the senior adulthood cohort. In Fig. 3, there were transitions towards worse outcome when CPP went below CPPopt in all age groups. The “optimized dichotomy” chiefly occurred between mortality and survival, the optimal GMT dichotomization was higher (10%) for ΔCPPopt ± 20 mmHg but lower for values outside this range, and the majority of patients were below the %GMT dichotomization point for negative ΔCPPopt in the youth and early adulthood and the middle adulthood group (Supplementary Fig. 1). Similar to PRx, these explanatory heatmaps indicate that ΔCPPopt had the strongest outcome association when the subgroup who died and exhibited a higher GMT burden with negative ΔCPPopt was analysed in relation to the remaining patients. These heatmaps differed in the senior adulthood group, particularly the outcome dichotomization occurred at around GOS 3 for negative ΔCPPopt but at GOS 1 for positive ΔCPPopt, while the percentage of patients below the GMT threshold remained around 25–50% throughout the ΔCPPopt range.

**Fig. 4:**
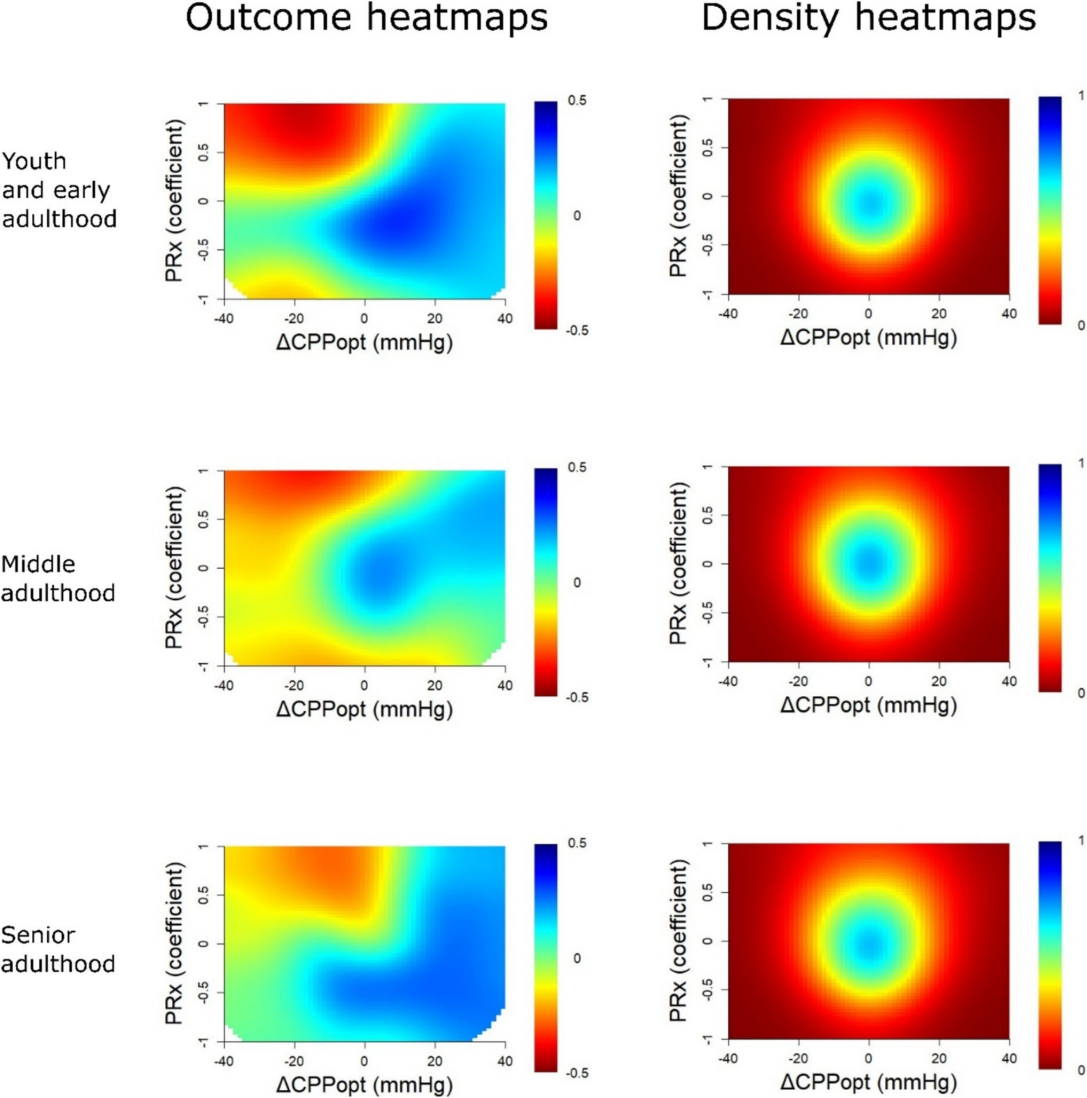
PRx/ΔCPPopt combinations – outcome and density heatmaps for all age groups. ***Outcome heatmaps*** – These figures show the correlation between the %GMT of PRx/ΔCPPopt combinations in relation to outcome. As demonstrated, there was a consistent trend towards worse outcome for high PRx in combinations with negative ΔCPPopt for all age groups. ***Density heatmaps*** – These figures show the data distribution of PRx/ΔCPPopt combinations for all age groups. CPPopt = Optimal cerebral perfusion pressure. GMT = Good monitoring time. PRx = Pressure reactivity index

**Fig. 5:**
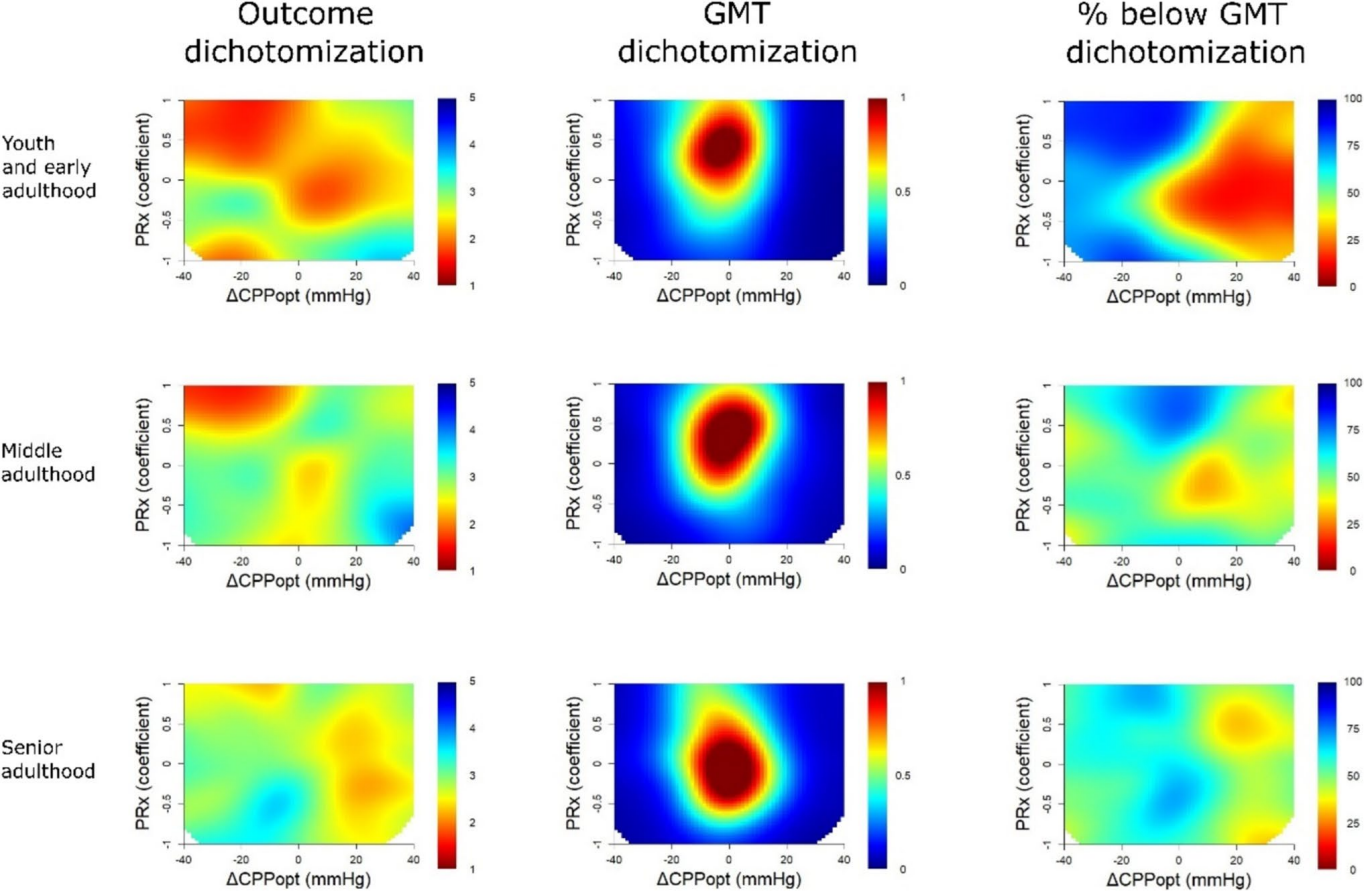
Dichotomizations points in the two-variable %GMT analysis of PRx/ΔCPPopt. ***Outcome dichotomization***– these heatmaps illustrate the dichotomization point in GOS for PRx/ΔCPPopt. As illustrated, the dichotomization typically occurred for mortality (GOS = 1) for the combination of high PRx and negative ΔCPPopt in the youth and middle adulthood group, while the same dichotomization occurred for the combination of lower PRx and more positive ΔCPPopt in the senior adulthood group. ***GMT dichotomization*** – these heatmaps illustrate the dichotomization point in %GMT for PRx/ΔCPPopt. As illustrated, the dichotomizations were typically made when the patients spent a relatively larger (red ≈ 1%) %GMT for PRx around 0 ± 0.50 and ΔCPPopt ± 10 mmHg for all age groups. ***% below GMT dichotomization*** – these heatmaps illustrate the percentage of patients below the GMT dichotomization point for PRx/ΔCPPopt. As illustrated, most patients were below the GMT dichotomization threshold for negative ΔCPPopt, while the concurrent PRx value had less influence on this variable. CPP = Cerebral perfusion pressure. CPPopt = Optimal CPP. ΔCPPopt = Actual CPP – CPPopt. GMT = Good monitoring time. PRx = Pressure reactivity index

**Fig. 6:**
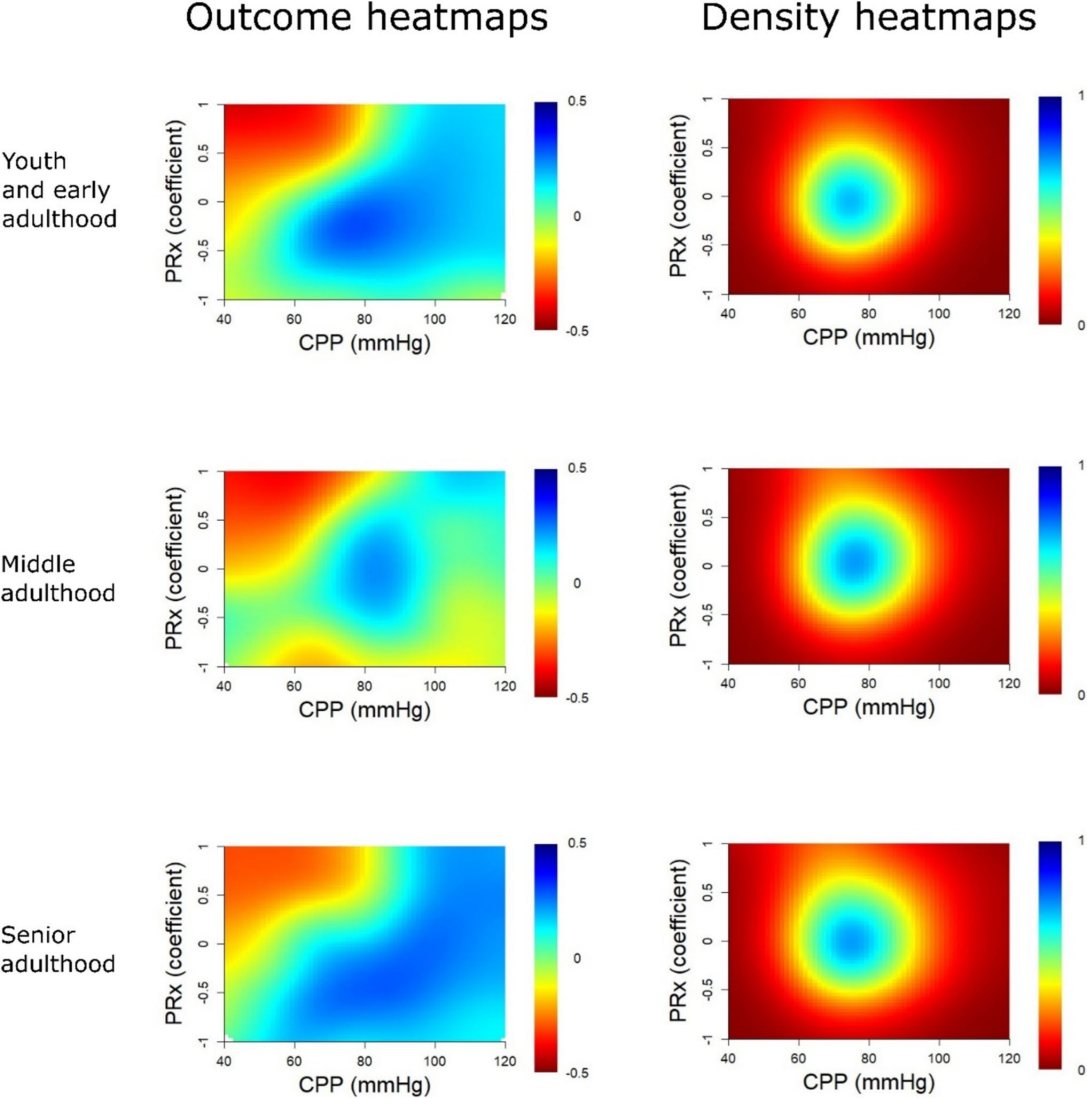
PRx/CPP combinations – outcome and density heatmaps for all age groups. ***Outcome heatmaps*** – These figures show the correlation between the %GMT of PRx/CPP combinations in relation to outcome. As demonstrated, there was a consistent trend towards worse outcome for high PRx in combinations with lower CPP for all age groups. ***Density heatmaps*** – These figures show the data distribution of PRx/CPP combinations for all age groups. CPP = Cerebral perfusion pressure. PRx = Pressure reactivity index

In Fig. 4, the combination of high PRx with negative ΔCPPopt was associated with lower GOS for all age categories. The “optimized dichotomy” chiefly occurred between mortality and survival for the combination of high PRx and negative ΔCPPopt, the optimal GMT dichotomization was higher (1%) for ΔCPPopt ± 20 mmHg with PRx above zero, and the majority of patients were below the %GMT dichotomization point for negative ΔCPPopt and high PRx in the youth and early adulthood and the middle adulthood group (Fig. 5). The senior adulthood group differed in some of these dichotomization patterns, particularly the outcome dichotomization appeared to occur between mortality and survival for positive ΔCPPopt for most of the PRx range. In Fig. 6, the combination of high PRx with negative ΔCPPopt was associated with lower GOS for all age categories. Overall, the “optimized dichotomy” occurred between survival and mortality for low CPP and high PRx, although such episodes were rare (< 1%), and most patients were above these GMT dichotomization thresholds (Supplementary Fig. 2).

## Discussion

In this large cohort of TBI patients, impaired CA with high PRx remained an independent predictor of worse outcome prognosis also in the senior adulthood cohort. When interactions with CPP and ΔCPPopt were examined using the two-variable heatmaps, PRx was helpful to determine the safe and dangerous CPP and ΔCPPopt intervals for all age groups, particularly in regard to the lower limit of CA with low CPP/negative ΔCPPopt and high PRx. Thus, according to our results, higher age, per se, should not necessarily exclude patients from future trials on CA-guided management.

Consistent with previous studies [3, 7, 15, 30], we found that TBI patients in senior adulthood exhibited worse CA with higher PRx-values compared to young patients. The senior adulthood patients also had a lower ICP, while CPP was comparable to the other age groups, indicating that intracranial hypertension and arterial hypotension did not explain these discrepancies. The underlying aetiology for higher PRx in elderly TBI patients remains to be determined in greater detail in future work, but it likely reflected differences in vascular ageing [12, 15] and TBI subtypes (focal vs. diffuse injury patterns) [36]. These aspects could not be explored in greater detail in this cohort and require further investigation in future studies.

Furthermore, our results showed that PRx remained an important independent predictor of outcome, particularly mortality, for all age groups. The single-variable heatmaps illustrated clear transitions towards lower GOS for higher PRx in all cases. The transition point varied slightly between the age groups and seemed to occur at lower PRx for the senior adulthood patients. These differences were most likely partly related to confounders, such as co-morbidities, TBI subtypes, and the burden of intracranial hypertension. The number of patients in each age group was also relatively limited and interpretations should therefore be made cautiously. However, it could be speculated that elderly and frail patients are less resilient to CA impairment with PRx elevations due to a reduced homeostatic capacity. There was also a trend towards worse outcome for negative ΔCPPopt in all age groups, but to a lesser extent among the senior adulthood patients for whom it did not reach significant levels in the statistical tests. However, the latter cohort was only based on 105 patients, which might not have been large enough to detect such an outcome association. In comparison, Lenell et al. [15] also found that higher PRx was independently associated with mortality, but not favourable recovery, while ΔCPPopt had no independent outcome association, in a cohort of elderly TBI patients from Uppsala. In that study, while high PRx together with both low CPP or negative ΔCPPopt (ischaemia pattern) and high CPP or positive ΔCPPopt (hyperaemia pattern) were particularly associated with worse outcome in the younger cohort, PRx provided almost no differentiation of safe and dangerous CPP or ΔCPPopt intervals in the cohort of elderly patients. In contrast, our results showed a consistent pattern among all age categories that PRx could aid to identify safe CPP and ΔCPPopt intervals. Specifically, lower PRx, indicating preserved CA, increased the tolerance for low CPP and negative ΔCPPopt. Therefore, the Uppsala’s results notwithstanding, our findings support that the CA variables, PRx and CPPopt, remain relevant as potential treatment targets also for elderly TBI patients.

It is intriguing that the interaction between the CA status vs. CPP and ΔCPPopt differed between the two centres, despite comparable numbers of patients. The Cambridge findings indicate that higher CPP seems better and positive ΔCPPopt is usually safe while negative values seem detrimental. In contrast, the Uppsala findings would argue for “the middle way”, or the Lund concept [11], actively avoiding both low and high CPP or keeping CPP close to CPPopt, particularly in case of CA impairment in younger patients. These discrepancies make it challenging to make any general recommendations regarding CPP or CPPopt targets that would be valid for all NCCUs. Therefore, it is necessary to further elucidate the reason for the discordant results between centres. Patient demography with differences in patient age among cohorts was previously considered as one plausible explanation [31], since younger patients might better maintain an intact CA at higher perfusion pressures and therefore tolerate high CPP better [20]. However, this study contradicts that idea, since there was no difference in the PRx vs. CPP or CPPopt plots among the age groups. Some alternative explanations would be that the underlying TBI subtype (focal vs diffuse injuries [27, 36]) or the management strategies (CPP targets and inclusion of CA metrics for clinical-decision-making) that were used influenced the associations between these CA variables and outcome. In future work, we aim to explore these issues in the multicentre CENTER-TBI cohort, in which such pathology descriptive data will be available.

Lastly, the “optimized dichotomy” heatmaps identified that the strongest outcome association for high PRx and negative ΔCPPopt were found when GOS was divided into mortality and survival and when the GMT was dichotomized at a relatively high %GMT around 10%, which seemed to capture a small proportion of the patients who exhibited significant detrimental, secondary, ischaemic brain injury. These visualizations are highly consistent with the formal statistical tests, including the multivariate logistic regressions, which showed that higher PRx and negative ΔCPPopt were particularly associated with mortality, but to a lesser extent or not at all with unfavourable outcome. Interestingly, the youth and early adulthood and the middle adulthood groups seemed to exhibit similar “optimized dichotomization” patterns, while the senior adulthood cohort differed in some ways. Particularly, the PRx and ΔCPPopt insults were to a greater extent dichotomized based on favourable and unfavourable outcome (GOS 4 to 5 vs. 1 to 3) in the latter group. These discrepancies may partly be explained by differences in prognostic variables, co-morbidities, and burden of secondary insults among the different age groups, but it also suggests that the type of gain in patient recovery, e.g., from mortality to survival or unfavourable to favourable outcome, by optimizing these autoregulatory targets may differ between the age groups.

### Strengths and novelty

While the association between higher PRx and increased age has been demonstrated in prior studies [3], the question of whether PRx provides prognostic information in different age categories in TBI has received limited attention [15, 21]. This study addresses this important gap by leveraging a large cohort of TBI patients with high-frequency physiological monitoring data. Additionally, we also used novel visualization methods [25, 27] of CA insults that allowed for granular threshold analyses and explorations of the interaction between PRx with CPP and ΔCPPopt.

### Limitations

Further co-morbidities and frailty data were not available in the Brain Physics database, thus, our analyses were limited to studies on chronological, rather than biological, age. Furthermore, we lacked data on TBI subtypes, which might have differed between the age groups and could have influenced the association between CA and outcome to some extent [15, 27]. Moreover, this study was conducted in a single-centre cohort of TBI patients deemed salvageable who were considered for ICP monitoring, introducing a selection bias. Patients with too mild injuries or perceived as too frail and/or with too dismal prognosis were excluded, limiting the generalizations to a subset of the senior adult TBI population. However, the patients were managed according to international guidelines, which partially mitigates this bias, but, inevitably, institutional protocols and clinician preferences influenced treatment decisions. Future studies should include more diverse populations and multi-center data to provide a broader understanding of optimal management strategies for senior TBI patients. Also, the study spanned over 20 years and both the patient demography and NCCU management slightly changed over this period. Particularly, the rate of elderly TBI patients increased and the reference point for the ABP/CPP was altered, which influenced CPP and CPPopt levels [9]. Thus, our findings should be interpreted cautiously. Still, the main variable of interest, PRx, and CPP deviation from CPPopt were not affected by this, and therefore we think our analyses remain valid. In addition, the reliability of PRx and CPPopt has sometimes been questioned following decompressive craniectomy, but preliminary studies [33] suggest that it remains preserved in this scenario, and we therefore abstained from excluding these patients from the analyses. The fact that PRx and CPPopt were available bedside from 2002 and 2012 respectively, potentially influencing management (albeit not in a protocolised way), also introduces a degree of bias that cannot be estimated from out data and thus might have impacted on their relationship with outcome. Furthermore, as indicated in the density heatmaps, the amount of data was small for certain physiological intervals and the association between %GMT and outcome should be analysed cautiously in these areas. Lastly, although the optimized dichotomization method was designed to highlight the relation between worsening of autoregulatory insults and outcome and to identify certain physiological and outcome clusters, these figures are occasionally challenging to interpret. These heatmaps are under constant technical development and will likely be more refined for greater interpretational impact, but this will only be possible for much larger datasets.

## Conclusions

In this large cohort of age-stratified TBI patients with high-frequency cerebral physiological data, impaired CA with high PRx remained an independent predictor of worse outcome prognosis for all age categories, including senior adulthood. It was also clear that the visualisation of interaction of PRx with CPP/ ΔCPPopt was helpful in determining the safe and dangerous CPP and ΔCPPopt intervals. Particularly, lower CPP and negative ΔCPPopt could be better tolerated when PRx was low. Considering that PRx and CPPopt also carry prognostic information in senior TBI patients, we suggest that higher age, per se, should not automatically exclude patients from future trials on autoregulatory-guided management.

## Supplementary Information

Below is the link to the electronic supplementary material.

ESM 1 (DOCX 353 KB)

ESM 2 (DOCX 14.6 KB)

## Data Availability

Data are available upon reasonable request.
