# Supplementary material for: Autoregulatory-guided management in traumatic brain injury: does age matter?
Source: Acta Neurochir (Wien). 2025 Feb 28;167(1):55. doi: 10.1007/s00701-025-06474-y (PMC11868309; doi:10.1007/s00701-025-06474-y)
Supplement: Supplementary file 1 — (DOCX 353 KB) [file 701_2025_6474_MOESM1_ESM.docx]

**Supplementary figure 1.** **Dichotomizations points in the single-variable %GMT analysis of ΔCPPopt**


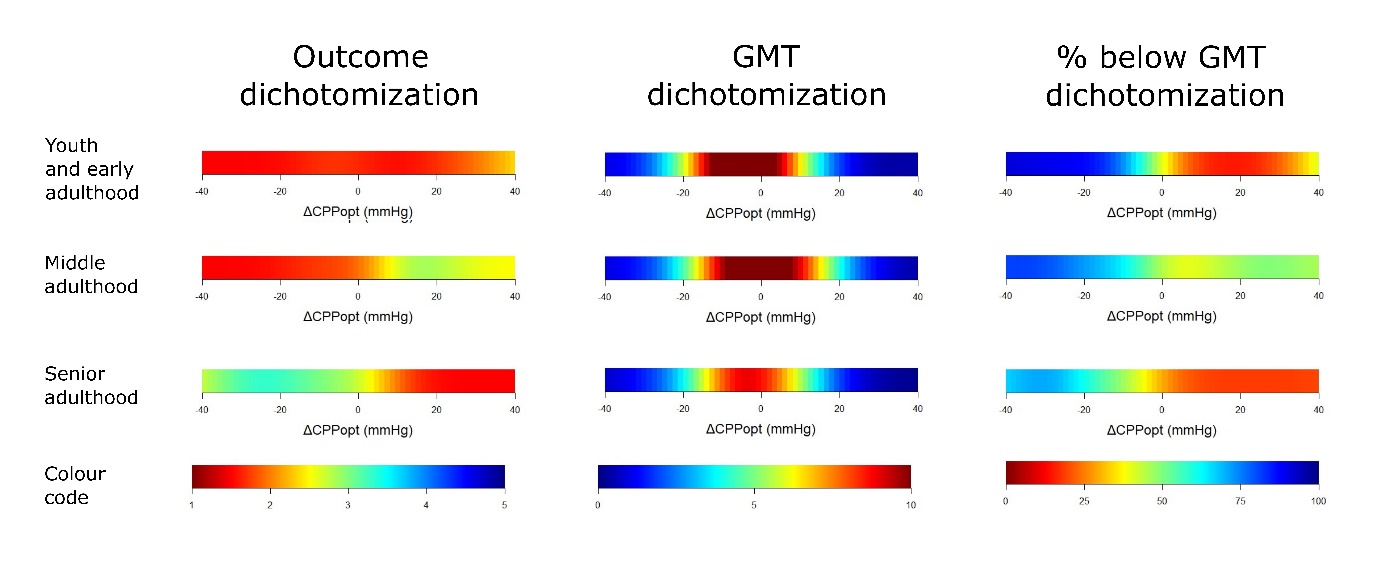


***Outcome dichotomization***– these heatmaps illustrate the dichotomization point in GOS for ΔCPPopt. As illustrated, the dichotomization typically occurred for mortality to severe disability over the entire ΔCPPopt range for the youth and early adulthood and the middle adulthood group, while it occurred around GOS 4 to 5 for negative ΔCPPopt and GOS 1 to 2 for positive ΔCPPopt for the senior adulthood group.

***GMT dichotomization*** – these heatmaps illustrate the dichotomization point in %GMT for ΔCPPopt. As illustrated, the dichotomizations were typically made when the patients spent a relatively larger (red ≈ 7-10%) %GMT for ΔCPPopt +20 mmHg and relatively smaller (blue < 5%) %GMT outside this range for all age groups.

***% below GMT dichotomization*** – these heatmaps illustrate the percentage of patients below the GMT dichotomization point for ΔCPPopt. As illustrated, most patients were below the GMT dichotomization threshold for positive ΔCPPopt and vice versa for negative values in the youth and early adulthood and the middle adulthood group, while it was distributed around 50% throughout the entire ΔCPPopt -range in the senior adulthood group.

CPP = Cerebral perfusion pressure. CPPopt = Optimal CPP. ΔCPPopt = Actual CPP – CPPopt. GMT = Good monitoring time.

**Supplementary figure 2.** **Dichotomizations points in the two-variable %GMT analysis of PRx/CPP**


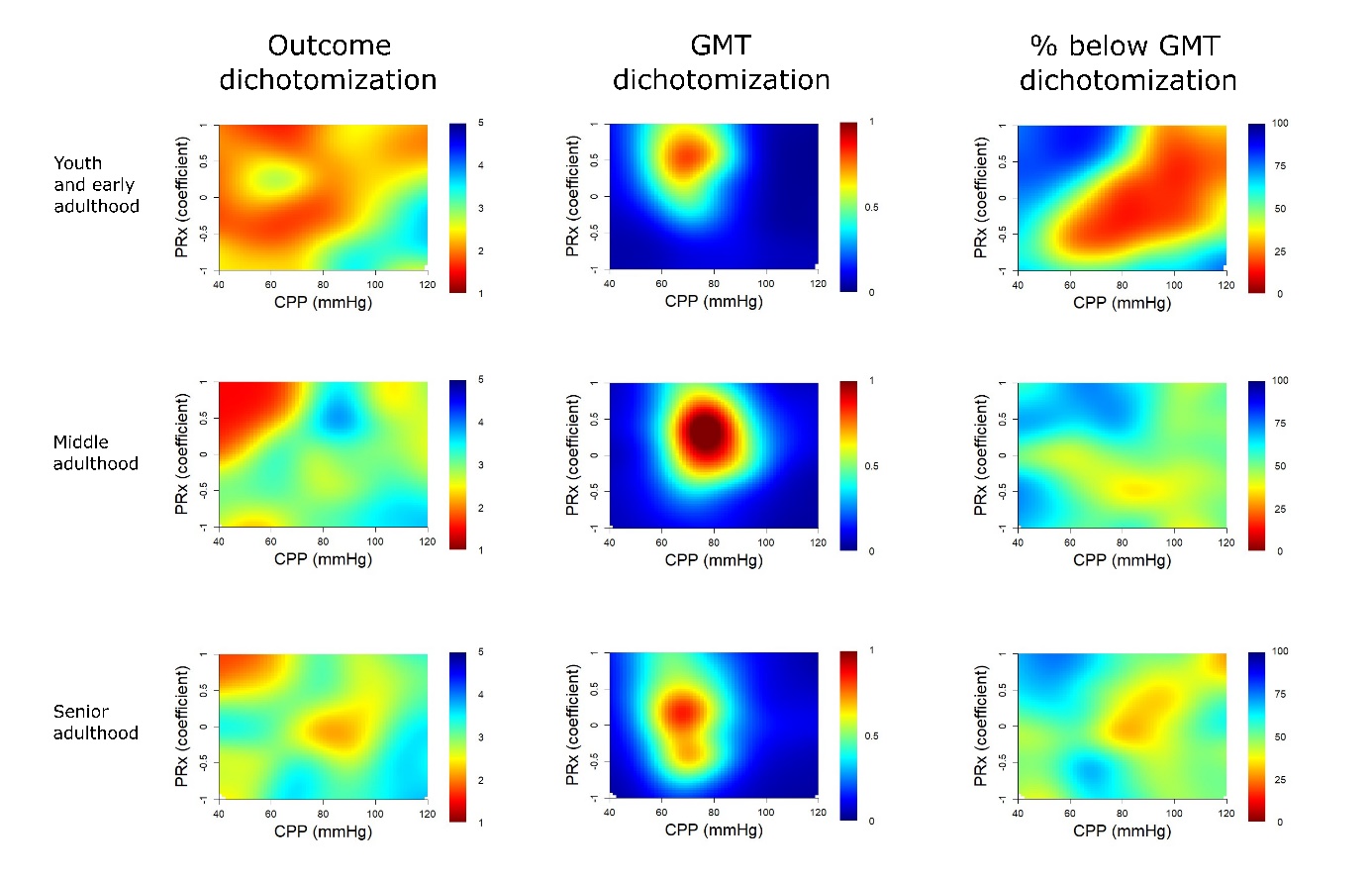


***Outcome dichotomization***– these heatmaps illustrate the dichotomization point in GOS for PRx/CPP. As illustrated, the dichotomization typically occurred for mortality (GOS = 1) for the combination of high PRx and lower CPP in the youth and early adulthood and the middle adulthood group. The senior adulthood group had a similar pattern, but the outcome dichotomizations were more varied over the PRx/CPP intervals.

***GMT dichotomization*** – these heatmaps illustrate the dichotomization point in %GMT for PRx/CPP. As illustrated, the dichotomizations were typically made when the patients spent a relatively larger (red ≈ 1%) %GMT for PRx at 0 ± 0.50 and CPP within 60 to 80 mmHg for all age groups.

***% below GMT dichotomization*** – these heatmaps illustrate the percentage of patients below the GMT dichotomization point for PRx/CPP. As illustrated, most patients were below the GMT dichotomization threshold for high PRx and low CPP, while it was relatively more common to be above the dichotomization point for the combination of higher PRx and high CPP in all age groups.

CPP = Cerebral perfusion pressure. GMT = Good monitoring time. PRx = Pressure reactivity index.
