# Supplementary material for: Autoregulatory-guided management in traumatic brain injury: does age matter?
Source: Acta Neurochir (Wien). 2025 Feb 28;167(1):55. doi: 10.1007/s00701-025-06474-y (PMC11868309; doi:10.1007/s00701-025-06474-y)
Supplement: Supplementary file 2 — (DOCX 14.6 KB) [file 701_2025_6474_MOESM2_ESM.docx]

**Supplementary table 1. Patients and cerebral physiological variables before and after the change of reference point of the blood pressure monitoring.**

|  | **Young** | | **Middle age** | | **Old** | |
| --- | --- | --- | --- | --- | --- | --- |
| **Variables** | **2002-2014** | **2015-2022** | **2002-2014** | **2015-2022** | **2002-2014** | **2015-2022** |
| Patients, n | 216 | 59 | 117 | 53 | 60 | 45 |
| ICP (mmHg), median (IQR) | ***16 (12-19)^c^*** | ***11 (9-14)^c^*** | ***15 (12-17)^c^*** | ***11 (8-14)^c^*** | ***15 (11-19)^c^*** | ***9 (7-12)^c^*** |
| CPP (mmHg), median (IQR) | ***76 (73-79)^c^*** | ***73 (70-76)^c^*** | ***78 (75-82)^c^*** | ***74 (71-78)^c^*** | ***75 (71-81)^a^*** | ***72 (69-78)^a^*** |
| PRx (coefficient), median (IQR) | +0.01 (-0.09-+0.13) | +0.02 (-0.07-+0.10) | +0.11 (-0.02-+0.24) | +0.04 (-0.05-+0.07) | +0.10 (-0.03-+0.24) | +0.10 (-0.01-+0.20) |
| CPPopt (mmHg), median (IQR) | ***76 (72-79)^c^*** | ***73 (69-75)^c^*** | ***78 (74-82)^b^*** | ***74 (71-78)^b^*** | ***75 (72-81)^b^*** | ***74 (71-76)^b^*** |

ABP was measured at heart level between 2002 and 2014 and at the foramen of Monro since 2015. The cerebral physiological variables were compared before and after this change for each age category. Superscripted letters indicate statistical significance; ^a^p < 0.05, ^b^p < 0.01, ^c^p < 0.001, which all were highlighted with bold and italics.

The proportion of young patients decreased between the early (2002-2014) and the late (2015-2022) period from 216 (55%) to 59 (38%), while the proportion of old patients increased from 60 (15%) to 45 (29%) and the middle-aged group increased slightly from 117 (30%) to 53 (34%).

ABP = Arterial blood pressure. CPP = Cerebral perfusion pressure. CPPopt = Optimal CPP. ICP = Intracranial pressure. IQR = Interquartile range. PRx = Pressure reactivity index.
